# Influence of age at slaughter and sex on carcass characteristics and technological and sensory quality of Goliath chicken meat

**DOI:** 10.14202/vetworld.2023.194-203

**Published:** 2023-01-28

**Authors:** A. G. A. Christie Ahokpossi, A. Gabriel Bonou, Kokou Tona, Issaka Youssao Abdou Karim, Yaovi Ameyapoh

**Affiliations:** 1 Regional Center of Excellence on Avian Sciences, University of Lomé, Lomé, Republic of Togo; 2 Laboratory of Animal Biotechnology and Meat Technology, University of Abomey-Calavi, Abomey-Calavi, Republic of Benin; 3 Laboratory of Microbiology and Food Quality Control, University of Lomé, Lomé, Republic of Togo

**Keywords:** Benin, carcass, goliath chicken, meat, quality

## Abstract

**Background and Aim:**

The Goliath chicken is a slow-growing chicken with a high slaughter weight but whose carcass characteristics and meat quality have not yet been documented. This study aimed to evaluate the effect of age at slaughter and sex on the carcass characteristics and technological and sensory meat quality of Goliath chickens raised in southern Benin.

**Materials and Methods:**

Data on the carcass characteristics and technological and sensory quality of meat were collected from 80 chickens raised in confinement and divided into two groups. The first group was reared for 12 weeks of age and the second group was for 20 weeks of age. The animals were individually weighed using an electronic balance (Terraillon 5000g) and then slaughtered. The different parts of the chicken carcass was weighed using the same method.

**Results:**

The live, hot carcass, cold carcass, wing, thigh, and tail weights of males were significantly greater than those of females (p < 0.01). The wishbone muscle and thigh and tail assembly pH was lower in the 20-week-old chickens than in the 12-week-old chickens. The brightness and red index of the wishbone of the 20-week-old chickens were significantly higher than those of the 12-week-old chickens (p < 0.001). The red index of the wishbone and the yellow index of the thigh and tail assembly of females were lower than those of males.

**Conclusion:**

The meat of 20-week-old Goliath chickens was juicier than that of 12-week-old chickens. Thus, the carcass composition of Goliath chickens is better at 20 weeks of age, especially in males.

## Introduction

Poultry farming is the first choice for national production intensification in Benin because of its short production cycle. Among the poultry species, local chickens provide the most meat for Beninese consumption [1]. The global chicken meat production was estimated to be 114,267 tons in 2018 [2]. The national Beninese chicken meat production was estimated to be 15 tons in 2018 for a population of 11,362,269 [3]. The local chickens that contribute the most to this production are slow-growing birds with a low live weight (1.2 kg on average) at maturity and a lean carcass [4]. Local chickens are raised primarily for meat production in small-scale traditional or improved farming systems. Despite the importance of poultry, national chicken production remains below the population demand. As a result, poultry meat imports increased three-fold the national production from 2008 to 2018 and from 94,084 to 117,511 tons [5]. However, consumers prefer local chickens compared to imported frozen chickens [6, 7].

In Benin, two main types of poultry farming are practiced: village poultry farming, based on the breeding of local chickens following an extensive system, and “modern” poultry farming, based on the breeding of imported breeds. Compared to exotic strains, local African chickens are quite hardy, allowing them to survive in harsh village or rural conditions without requiring special care [8]. They are good brooders and excellent mothers, but are known for their slow growth, late egg laying, and low productivity with varying levels depending on the region and rearing conditions, including feed composition. Unlike other local chicken populations of the North, South, Fulani, and Sahoue ecotypes [9], the carcass characteristics and meat quality of the Goliath chicken have not yet been studied. These characteristics may be influenced by factors such as sex, age, and rearing conditions of the birds.

Thus, this study aimed to evaluate the influence of factors, mainly age at slaughter and sex, on the characteristics of the Goliath chicken carcass and its meat quality.

## Materials and Methods

### Ethical approval

The animals were raised and slaughtered in accordance with the requirement of the Ethic Committee in production and animal health following the reference No. 025/LBATV/LARBA/EPAC/UAC dated 04 October 2021 of Laboratory of Animal Biotechnology and Meat Technology of the University of Abomey-Calavi.

### Study period and location

The study was conducted from October 2021 to February 2022. The Goliath chickens used in this study were raised in southern Benin, specifically in the avian experimentation farm of the Laboratory of Animal Biotechnology and Meat Technology of the Polytechnic School of Abomey-Calavi of the University of Abomey-Calavi. This farm is located in the commune of Abomey-Calavi, Togba district in the Agori region, at 6°42’6” North longitude and 2°32’4” West latitude. The commune of Abomey-Calavi is bounded to the North by the commune of Ze, to the South by the Atlantic Ocean, to the East by the communes of So-Ava and Cotonou and to the West by the communes of Tori-Bossito and Ouidah. It has an area of 539 km^2^ and a population of 1434544 inhabitants in 2019 [3]. The climate is sub-equatorial, with two rainy seasons and two dry seasons. The long rainy season begins in April and ends in July and the short dry season lasts from August to mid-September. This is followed by the short rainy season, which runs from September to early November, and the long dry season, which lasts from early December to March.

### Animal husbandry

The study was conducted on 80 Goliath chickens (40 males and 40 females) from a core group of breeders acquired from Goliath chicken breeders in southern Benin. These chickens were reared in confinement and fed the same diet until they were 12 weeks old, when the first batch (20 males and 20 females) was slaughtered. The second batch was reared to 20 weeks of age before slaughter. Three types of feed were fed to the chicks during the rearing period. The complete diets used in the experiment were purchased from Groupe Veto Service (GVS), for each age category and their characteristics are presented in Table-1.

**Table-1:** Nutritional composition of the diets.

Constituents	Starter	Grower	Finisher
Crude protein mater (%)	21	19.03	16
Lysine (%)	1.15	0.97	0.8
Methionine (%)	0.6	0.45	0.4
Calcium (%)	1.03	1	0.96
Total phosphorus (%)	0.55	0.5	-
Crude ash (%)	7.78	7.48	6.5
Crude cellulose (%)	4.32	4.6	4.47
Crude fat (%)	5.77	5	4.8
Metabolizable energy (kcal/kg)	2800	2800	2500

Source: Feed bag tag (Groupe Véto Service SA)

### Health monitoring

To ensure the health protection of the birds, they were subjected to a prophylactic plan (vaccination coverage, internal and external deworming, antibiotic therapy, vitamins, and anti-stress) (Table-2). Thus, day-old chicks and pullets were vaccinated against Newcastle disease, Gomboro disease and infectious bronchitis. The vaccines used during the experiment were CEVAC New L, Bronipra, CEVAC Gumbo L, Hiprapox, Ita New and CEVAC IBIRD. Preventive treatment was also applied for coccidiosis (oral); the drugs used were Amprolim^®^ (Laprovet) for 5 days, and Anticox^®^ (Laprovet, Tours, France) for 3 days. The birds were dewormed every month; an anti-stress medication was used for the animals for a few days according to the prophylactic plan. The litter was changed thrice during the rearing period.

**Table-2:** Prophylaxis plan used.

Age		Medical prophylaxis	Products

Weeks	Days		
1	1	Anti-stress	Sweetened water
	2	Newcastle disease vaccine + Bronchitis + Anti-stress after vaccine	Hipraviar B1 L + Bronipra + Alfaceryl
	3	Gumboro disease vaccine + Anti-stress	CEVAC Gumbo L + Alfaceryl
	4–7	Anti-stress	Alfaceryl
2	10	Reminder Newcastle disease vaccine + Bronchitis + Anti-stress after vaccine	CEVAC New L + Bronipra + Alfaceryl
	11	Reminder Gumboro disease vaccine + Anti-stress	CEVAC Gumbo L + Alfaceryl
	12–14	Anti-stress	Tetracolivit
3	15–17	Anticoccidial	Anticox
	18–20	Anti-stress	Tetracolivit
4	28	Dewormer	Piperazine
5	29–31	Anti-stress	Tetracolivit
	32–35	Hepatoprotector	Vigosine
6	36	Hepatoprotector	Vigosine
	38–42	Vitamin	Amin’ total
7	45	Smallpox vaccine + Anti-stress anfter vaccine	Hiprapox + Tetracolivit
	46–47	Anti-stress	Alfacryl
	48	Newcastle Disease + Anti-stress after vaccine	Itanew
	49–50	Anti-stress	Alfacryl
8	51–53	Anticoccidial	Anticox
	54–56	Anti-stress	Alfaceryl
	57	Dewormer	Piperazine
9	58–60	Anti-stress	Alfaceryl
10	70	Reminder vaccine of Newcastle Disease + Anti-stress after vaccine	Ita New (injectable) + Tetracolivit
11	71–74	Anti-stress	Tetracolivit
	75	Infectious bronchitis vaccine + Anti-stress after vaccine	CEVAC IBIRD + Alfaceryl
	76–77	Anti-stress	Tetracolivit
12	78–79	Anti-stress	Tetracolivit
	84	Dewormer	Piperazine
13	85–87	Anti-stress	Tetracolivit
16	112	Dewormer	Piperazine
17	113–115	Anti-stress	Tetracolivit
18	120–124	Anticoccidial	Amprolium
	125–126	Anti-stress	Tetracolivit
19	133	Dewormer	Piperazine
20	134–138	Anti-stress	Tetracolivit

### Slaughter and body composition of chickens

At the end of the 12 weeks, the chickens were divided into two batches that included 20 males and 20 females per batch to ensure that the batches were homogeneous, and one batch was slaughtered. At week 20, the second batch was slaughtered. The live weight of the birds was measured using an electronic scale with a capacity of 7000 ± l g. A 12 h water diet was observed before slaughter. After bleeding, the chickens were scalded in 75°C water and manually plucked. The legs were severed at the tarsometatarsal joint, and the head was separated from the neck at the skull-atlas junction. The organs of the abdominal and thoracic cavities (viscera) were removed. The hot carcasses were weighed and placed in sterile bags in a cooler containing dry ice for transport to the laboratory and then stored for 24 h.

### pH measurement

Each day, pH measurements were performed using a HANNA portable pH meter which was previously calibrated with two pH standards, at pH 4 and 7, according to the procedure described by the manufacturer (HANNA Instruments R, Italy). The pH measurements were performed on the right slice of the Pectoralis major (PM) muscle (brevis muscle) and in the *Iliotibialis superficialis* muscle of the right thigh. At each measurement timepoint, five replicates were performed per slice. The pH values were measured at 1 and 24 h after slaughter.

### Color determination of the chicken meat

The color of the meat was determined according to the standards of the Commission Internationale d’Eclairage (1978) after the samples were exposed to the ambient air for 90 min under film. The color was measured 24 h after slaughter and measurements were taken from the ventral side of the upper third of the left slice of the PM muscle at its thickest part and on the ventral side at the central level of the *Iliotibialis superficialis* muscle of the left thigh, using a chroma meter. The color indices measured included luminosity L*, red index a*, and yellow index b*. The saturation or chromaticity (C) and hue (h) were determined according to the formulas:

C = (a*2+b*2) “2

H = tan ‘ b*/a*.

### Determination of water retention capacity

Fifty grams of the left slice of PM and *Iliotibialis superficialis* muscles from each chicken were used for the determination of juice loss by flow and cooking. Each sample was suspended from a hook and placed in a refrigeration bag without touching the bottom of the bag. After 24 h in the refrigerator in the hanging position, the sample was removed from the bag without touching the bottom which contained the drip juice. This was blotted and weighed. The juice loss by drainage in 24 h was calculated per sample and expressed as a percentage of the initial weight. The samples were then placed in cooking bags and heated in a water bath to 80°C. After cooking for 20 min, the samples were removed, cooled, blotted, and weighed. Baking loss was calculated and expressed as a percentage. The water retention capacity was obtained by adding the drip and baking loss.

### Sensory analysis of the chicken meat

The pH of the PM and *Iliotibialis superficialis* muscles of each chicken was measured and used for sensory analysis. A panel of 10 trained judges was included for the tasting assessment. Each sample was placed in a cooking bag without seasoning and cooked in a water bath to a core temperature of 75°C. The samples were allowed to cool at room temperature and each cooked meat sample was cut into ten identical pieces. Each judge was given one piece from each category and batch of chicken on a plate previously divided into four different colored parts. A total of four samples, two (one male and one female) per batch, were placed on the numbered plates (1–4) on adhesive papers that were glued separately to the plate so that two parallel rows of two samples each were obtained. The judges were instructed on the order of tasting for each sample and simultaneously evaluated the three sensory characteristics of tenderness, juiciness, and flavor. These characteristics were scored on a scale from 1 to 5. For tenderness, 1 corresponded to very hard, 2 to hard, 3 to acceptable, 4 to soft, and 5 to very soft. For the juiciness, 1 corresponded to very dry, 2 to dry, 3 to acceptable, 4 to juicy, and 5 to very juicy. For the intensity of the flavor, 1 corresponded to very weak, 2 to weak, 3 to acceptable, 4 to strong, and 5 to very strong. Finally, each of the judges assigned an overall score from 1 to 10. At the end of the tasting session, a form was completed to summarize the results.

### Statistical analysis

The collected data were analyzed using R software. The two-factor analysis of variance test was used. The factors of variation were age at slaughter and sex. The interaction between age at slaughter and sex was evaluated. The means and standard deviations of the studied variables were calculated and compared in pairs using the student t-test.

## Results

### Carcass characteristics of Goliath chickens by age at slaughter and sex

The carcass characteristics of Goliath chickens by age and sex are presented in Table-3. With respect to the age and live, hot carcass, cold carcass, wishbone, wing, thigh, and tail weights of 20-week-old chickens were significantly higher than those of 12-week-old chickens (p < 0.001). The same trend was observed for carcass yields and breast and thigh drumsticks proportions. Significant variations were also observed according to sex; the live weight of males (1925.25 ± 439.22 g) was significantly higher (p < 0.01) than that of females (1523.75 ± 479.46 g). The same was true for the hot carcass, cold carcass, wing, thigh, and tail weights, and the proportions of wishbone muscle and thigh and tail assembly. In contrast, no sex differences were observed in the wishbone weight and hot and cold carcass yields. The interaction between age at slaughter and sex was only significant for the proportion of wishbone, where females had a higher value than males at 20 weeks of age (p < 0.05).

**Table-3:** Body composition of Goliath chickens according to age at slaughter and sex.

Parameters	(Age at slaughter (mean ± SD)		Sex (mean ± SD)		12 Weeks (mean ± SD)		20 weeks (mean ± SD)		Significance test		
				
	12 weeks	20 weeks	Female	Male	Female	Male	Female	Male	Age	Sex	Age* Sex
Weight (g)	1305.5 ± 272.86^b^	2143.5 ± 252.16^a^	1523.75 ± 479.46^b^	1925.25 ± 439.22^a^	1079.9 ± 127.26^a^	1531.1 ± 167.00^a^	1967.6 ± 177.00^a^	2319.4 ± 184.78^a^	***	***	NS
Carcass h (g)	822.9 ± 198.07^b^	1532.7 ± 234.14^a^	1030.55 ± 397.75^b^	1325.05 ± 394.16^a^	662.1 ± 108.60^a^	983.7 ± 116.50^a^	1399.0 ± 143.23^a^	1666.4 ± 235.57^a^	***	***	NS
Carcass c (g)	815.30 ± 198.43^a^	1547.85 ± 230.70^b^	1035.65 ± 412.63^b^	1327.50 ± 399.92^a^	650.0 ± 92.45^a^	980.6 ± 117.71^a^	1421.3 ± 142.82^a^	1674.4 ± 237.43^a^	***	***	NS
Breast (g)	179.31 ± 44.56^b^	432.8 ± 61.29^a^	290.95 ± 155.16^a^	321.30 ± 122.32^a^	148.3 ± 32.55^a^	210.6 ± 31.25^a^	433.6 ± 67.44^a^	432.0 ± 67.44^a^	***	NS	NS
Wings	130.40 ± 34.72^b^	208.15 ± 63.68^a^	136.75 ± 47.04^b^	201.80 ± 63.27^a^	101.8 ± 16.35^a^	159.0 ± 21.46^a^	171.7 ± 41.10^a^	244.6 ± 62.61^a^	***	***	NS
Thigh-drumstick (g)	285.35 ± 74.55^b^	498.20 ± 104.23^a^	322.30 ± 112.57^b^	461.25 ± 132.04^a^	222.2 ± 29.75^a^	348.5 ± 44.59^a^	422.4 ± 60.00^a^	574.0 ± 81.04^a^	***	***	NS
Carcass h (%)	62.69 ± 3.96^b^	71.43 ± 5.94^a^	66.18 ± 7.21^a^	67.93 ± 6.l 2^a^	61.18 ± 5.03^a^	64.19 ± 1.63^a^	71.19 ± 5.37^a^	71.66 ± 6.75^a^	***	NS	NS
Carcass c (%)	62.07 ± 3.96^b^	72.16 ± 5.82^a^	66.24 ± 7.72^a^	67.99 ± 6.29^a^	60.16 ± 4.21^a^	63.98 ± 1.62^a^	72.32 ± 5.12^a^	72.00 ± 6.72	***	NS	NS
Breast (%)	21.80 ± 3.96^b^	28.58 ± 4.30^a^	26.60 ± 5.32^a^	23.79 ± 3.71^b^	22.24 ± 2.28^b^	21.36 ± 1.31^b^	30.95 ± 3.78^b^	26.21 ± 3.52^a^	***	**	*
Thigh-drumstick (%)	15.85 ± 1.90^a^	13.42 ± 2.73^b^	13.91 ± 2.99^b^	15.36 ± 2.03^b^	15.51 ± 2.30^a^	16.18 ± 1.45^a^	12.30 ± 2.80^a^	14.54 ± 2.27^a^	**	*	NS

* p < 0.05,

** p < 0.01,

*** p < 0.001, NS: P > 0.05. SD=Standard deviation; Means of the same line followed by different letters differ significantly at the 5% threshold, carcass h=Carcass hot, carcass c=Carcass cold

### Variation in meat pH of Goliath chickens with age at slaughter and sex

The variation of pH with age and sex are presented in Table-4. With respect to age at slaughter, at 1 h after slaughter, the pH of the thigh and tail assembly of the chickens slaughtered at 12 weeks was higher than that of the chickens slaughtered at 20 weeks (6.45 ± 0.11 vs. 6.41 ± 0.16). The same was true for the pH of the wishbone and thigh and tail assembly at 24 h after slaughter (5.98 ± 0.12; 6.17 ± 0.17 vs. 5.64 ± 0.17; 5.77 ± 0.13) (p < 0.001). Regarding sex, only the pH of the thigh and loins at 24 h after slaughter varied and was higher in males (6.01 ± 0.27) than in females (5.94 ± 0.21).

**Table-4:** Variations in meat pH of Goliath chickens by age at slaughter and sex.

Parameters	Organs	Age at slaughter (mean ± SD)		Sex (mean ± SD)		12 weeks (mean ± SD)		20 weeks (mean ± SD)		Significance test		
				
		12 weeks	20 weeks	Female	Male	Female	Male	Female	Male	Age	Sex	Age* Sex
pH 1h	Breast	6.37 ± 0.11^a^	6.51 ± 1.90^a^	6.56 ± 1.90^a^	6.32 ± 0.16^a^	6.38 ± 0.08^a^	6.36 ± 0.14^a^	6.74 ± 2.68^a^	6.29 ± 0.17^a^	NS	NS	NS
	Thigh-d	6.45 ± 0.11^a^	6.41 ± 0.16^b^	6.43 ± 0.13^a^	6.43 ± 0.15^a^	6.40 ± 0.09^b^	6.5 ± 0.11^a^	6.45 ± 0.17^a^	6.37 ± 0.15^b^	*	NS	***
pH 24 h	Breast	5.98 ± 0.12^a^	5.64 ± 0.17^b^	5.82 ± 0.21^a^	5.79 ± 0.24^a^	5.97 ± 0.15^a^	5.99 ± 0.09^a^	5.67 ± 0.16^b^	5.6 ± 0.18^b^	***	NS	NS
	Thigh-d	6.17 ± 0.17^a^	5.77 ± 0.13^b^	5.94 ± 0.21^b^	6.01 ± 0.27^a^	6.10 ± 0.14^a^	6.25 ± 0.16^b^	5.79 ± 0.15^b^	5.77 ± 0.11^b^	***	**	***

* p < 0.01,

*** p < 0.001, NS: P > 0.05. SD=Standard deviation, Means of the same line followed by different letters differ significantly at the 5% threshold, Thigh-d=Thigh-drumstick

In addition, the interaction between the age at slaughter and sex was significant at 12 weeks, with a higher pH value in males than in females (p < 0.001) in the thigh and tail muscles at both measurement time points. At 20 weeks, an inverse trend was observed in the pH of this muscle between the two sexes (p < 0.001).

### Variations in meat water holding capacity parameters of Goliath chickens by age at slaughter and sex

The parameters of water retention capacity of Goliath chicken meat are presented in Table-5. With respect to age, the water loss by muscle flow from the thigh and tail assembly of 12-week-old Goliath chickens (4.05 ± 2.68%) was significantly higher (p < 0.001) than that of 20-week-old chickens (2.27 ± 2.15%). No significant difference was observed for cooking water loss. The cooking water loss plus the run-off water loss of the thigh and tail assembly of chickens slaughtered at 20 weeks (23.99 ± 7.17) was lower than that of chickens slaughtered at 12 weeks (27.97 ± 5.35). Regarding sex, male carcasses had a higher cooking water loss than that of females. The same trend was observed in the chicken wishbone muscle for water-holding capacity. In addition, water loss by flow varied with age, and at 20 weeks of age, males had a higher water loss than females.

**Table-5:** Variations in meat water holding capacity parameters of Goliath chickens according to age at slaughter and sex (%).

Variables (%)	Organs	Age at slaughter (mean ± SD)		Sex (mean ± SD)		12 weeks (mean ± SD)		20 weeks (mean ± SD)		Significance Test		
				
		12 weeks	20 weeks	Female	Male	Female	Male	Female	Male	Age	Sex	Age*Sex
Drip loss	Breast	3.63 ± 3.06^a^	4.62 ± 5.39^a^	4.38 ± 3.39^a^	3.87 ± 5.22^a^	4.27 ± 3.76^a^	4.98 ± 6.82^a^	4.50 ± 3.16^a^	2.76 ± 2.82^b^	NS	NS	*
	Thigh-d	4.05 ± 2.68^a^	2.27 ± 2.15^b^	3.47 ± 2.89^a^	2.85 ± 2.21^a^	2.70 ± 2.27^a^	1.83 ± 2.03^a^	4.23 ± 3.34^a^	3.87 ± 1.96^a^	***	NS	NS
Cooking loss	Breast	18.43 ± 4.23^a^	19.82 ± 5.10^a^	16.93 ± 4.29^b^	21.32 ± 4.05^a^	16.81 ± 4.47^a^	22.82 ± 3.84^a^	17.05 ± 4.32^a^	19.82 ± 3.84^a^	NS	***	NS
	Thigh-d	23.91 ± 5.61^a^	21.72 ± 6.44^b^	21.04 ± 5.47^b^	24.59 ± 6.24^a^	16.81 ± 4.47^a^	22.82 ± 3.84^a^	17.05 ± 4.32^a^	19.82 ± 3.84^a^	*	*	NS
WHC	Breast	22.07 ± 3.64^a^	24.44 ± 8.27^a^	21.32 ± 5.17^b^	25.20 ± 7.07^a^	21.08 ± 6.28^b^	27.81 ± 4.87^a^	21.55 ± 4.21^b^	22.58 ± 3.09^b^	NS	*	*
	Thigh-d	27.97 ± 5.35^a^	23.99 ± 7.17^b^	24.51 ± 5.91^a^	27.45 ± 6.98^a^	23.86 ± 7.15^b^	24.12 ± 7.53^b^	25.15 ± 4.63^b^	23.78 ± 4.61^b^	*	NS	NS

* p < 0.05, **p < 0.01,

*** p < 0.001, NS: P > 0.05, Means of the same line followed by different letters differ significantly at the 5% threshold, Thigh-d=Thigh-drumstick, WHC=Water holding capacity

### Meat color of Goliath chickens by age at slaughter and sex

The meat color characteristics of Goliath chickens by age at slaughter and sex are presented in Table-6. Regarding age, the brightness and red index of the wishbone muscle of 20-week-old chickens were significantly higher than those of 12-week-old chickens (p < 0.01). In the thigh and tail muscles, the red index of the 20-week-old chickens was higher than that of the 12-week-old chickens (p < 0.05). An opposite trend was observed with the hue. As for sex, the red index and chromaticity of the wishbone muscle and the brightness of the thigh and tail assembly of males were higher than those of females (p < 0.001).

**Table-6:** Variations in meat color parameters of Goliath chickens by age at slaughter and sex.

Organs	Variables	Age at slaughter (mean ± SD)		Sex (mean ± SD)		20 weeks (mean ± SD)		12 weeks (mean ± SD)		Significance test		
				
		20 weeks	12 weeks	Female	Male	Female	Male	Female	Male	Age	Sex	Age*Sex
Breast	L*	58.95 ± 7.18^a^	54.18 ± 5.13^b^	57.02 ± 6.46^a^	56.11 ± 6.86^a^	58.95 ± 6.12^a^	58.94 ± 8.16^a^	55.09 ± 6.26^a^	53.27 ± 3.48^a^	***	NS	NS
	a*	5.86 ± 2.46^a^	4.69 ± 1.81^b^	4.54 ± 1.91^b^	6.01 ± 2.29^a^	4.82 ± 1.96^b^	6.90 ± 2.48^a^	4.25 ± 1.84^b^	5.13 ± 1.68^b^	***	***	*
	b*	5.69 ± 2.76^a^	5.19 ± 2.14^a^	5.25 ± 2.39^a^	5.62 ± 2.55^a^	5.28 ± 2.66^a^	6.09 ± 2.83^a^	5.22 ± 2.12^a^	5.16 ± 2.17^a^	NS	NS	NS
	C	8.34 ± 2.59^a^	7.18 ± 1.49^a^	7.14 ± 1.77^b^	8.38 ± 2.39^a^	7.32 ± 2.12^a^	9.36 ± 2.71^a^	6.95 ± 1.43^a^	7.40 ± 1.59^a^	NS	*	NS
	h	0.74 ± 0.18^a^	0.82 ± 0.20^a^	0.83 ± 0.21^a^	0.73 ± 0.17^a^	0.79 ± 0.16^a^	0.68 ± 0.20^a^	0.88 ± 0.24^a^	0.77 ± 0.12^a^	NS	NS	NS
Thigh-d	L*	45.64 ± 7.11^a^	46.91 ± 4.42^a^	44.70 ± 6.99^b^	47.84 ± 4.14^a^	43.48 ± 8.32^a^	47.80 ± 4.84^a^	45.92 ± 5.13^a^	47.89 ± 3.35^a^	NS	***	NS
	a*	18.29 ± 5.87^a^	16.05 ± 3.67^b^	17.56 ± 5.19^a^	16.79 ± 4.82^a^	18.40 ± 6.24^a^	18.18 ± 5.52^a^	16.71 ± 3.73^ab^	15.40 ± 3.53^b^	***	NS	NS
	b*	9.80 ± 6.10^a^	10.69 ± 2.77^a^	9.96 ± 3.07^a^	10.53 ± 5.98^a^	8.88 ± 2.91^b^	10.73 ± 8.06^a^	11.05 ± 2.86^a^	10.33 ± 2.66^a^	NS	NS	*
	C	21.13 ± 3.50^a^	19.36 ± 3.35^a^	20.35 ± 3.36^a^	20.14 ± 3.72^a^	20.58 ± 3.31^a^	21.67 ± 3.76^a^	20.12 ± 3.56^a^	18.60 ± 3.11^a^	NS	NS	NS
	h	0.48 ± 0.06^b^	0.58 ± 0.06^a^	0.51 ± 0.09^a^	0.55 ± 0.07^a^	0.45 ± 0.06^a^	0.51 ± 0.06^a^	0.58 ± 0.08^a^	0.59 ± 0.05^a^	***	NS	NS

* p < 0.1, **p < 0.01,

*** p < 0.001, NS: P > 0.05, Means of the same line followed by different letters differ significantly at the 5% threshold, L*=Lightness, a*=Red index, b*=Yellow index, C=Chromaticity, h=Hue

In addition, the interaction between age at slaughter and sex was significant, with the red index of the sternum muscle of males being higher than that of females at 12 and 20 weeks of age. In the thigh, the yellow index of female meat was lower than that of male meat at 20 weeks of age (p < 0.05).

### Variations in sensory parameters of Goliath chicken meat by age at slaughter and sex

The results of the sensory analysis of the meat of the wishbone and thigh and tail assembly of Goliath chickens as a function of age and sex are presented in Table-7. With respect to age at slaughter, the juiciness of the wishbone meat of 20-week-old chickens was higher than that of 12-week-old chickens. There were no observed variations between the rest of the sensory parameters of the meat (p > 0.05). The interaction between age at slaughter and sex was not significant.

**Table-7:** Variations in sensory parameters of Goliath chicken meat by age at slaughter and sex.

Parameter	Variables	Age (mean ± SD)		Sex (mean ± SD)		12 weeks (mean ± SD)		20 weeks (mean ± SD)		Significance test		
				
		12 weeks	20 weeks	Female	Male	Female	Male	Female	Male	Age	Sex	Age*Sex
Tenderness	Breast	3.38 ± 0.59^a^	3.59 ± 0.66^a^	3.51 ± 0.57^a^	3.46 ± 0.69^a^	3.40 ± 0.60^a^	3.36 ± 0.60^a^	3.62 ± 0.55^a^	3.56 ± 0.79^a^	NS	NS	NS
	Thigh-d	3.48 ± 0.60^a^	3.53 ± 0.58^a^	3.49 ± 0.54^a^	3.52 ± 0.64^a^	3.48 ± 0.51^a^	3.48 ± 0.70^a^	3.50 ± 0.59^a^	3.56 ± 0.60^a^	NS	NS	NS
Juiciness	Breast	3.12 ± 0.40^b^	3.59 ± 0.45^a^	3.41 ± 0.43^a^	3.30 ± 0.53^a^	3.24 ± 0.40^a^	3.0 ± 0.37^a^	3.58 ± 0.41^a^	3.6 ± 0.50^a^	**	NS	NS
	Thigh-d	3.17 ± 0.56^a^	3.35 ± 0.50^a^	3.24 ± 0.58^a^	3.28 ± 0.50^a^	3.12 ± 0.52^a^	3.22 ± 0.62^a^	3.36 ± 0.63^a^	3.34 ± 0.35^a^	NS	NS	NS
Flavor	Breast	3.09 ± 0.58^a^	3.47 ± 0.66^a^	3.35 ± 0.68^a^	3.21 ± 0.62^a^	3.2 ± 0.63^a^	2.98 ± 0.53^a^	3.5 ± 0.72^a^	3.44 ± 0.63^a^	NS	NS	NS
	Thigh-d	3.08 ± 0.77^a^	3.40 ± 0.61^a^	3.30 ± 0.59^a^	3.18 ± 0.81^a^	3.14 ± 0.49^a^	3.02 ± 1.0^a^	3.46 ± 0.66^a^	3.34 ± 0.58^a^	NS	NS	NS
Overall score	Breast	7.00 ± 0.93^a^	7.45 ± 1.05^a^	7.37 ± 0.97^a^	7.08 ± 1.03^a^	7.24 ± 0.95^a^	6.76 ± 0.89^a^	7.50 ± 1.03^a^	7.40 ± 1.11^a^	NS	NS	NS
	Thigh-d	6.60 ± 1.40^a^	7.04 ± 1.17^a^	6.89 ± 1.26^a^	6.75 ± 1.35^a^	6.72 ± 1.27^a^	6.48 ± 1.57^a^	7.06 ± 1.29^a^	7.02 ± 1.10^a^	NS	NS	NS

* p < 0.01, ***p < 0.001, NS: P > 0.05, Means of the same line followed by different letters differ significantly at the 5% threshold, Thigh-d=Thigh-drumstick

## Discussion

### Carcass characteristics of Goliath chickens by age at slaughter and sex

The live, hot carcass, cold carcass, wishbone, wing, thigh, and shank weights, as well as carcass yields and proportion of wishbone of 20-week-old chickens were significantly higher than those of 12-week-old chickens. Carcass weights as well as the yield of different parts of Goliath chickens changed significantly with age. This observation provides evidence that Goliath chickens continue to grow significantly between 12 and 20 weeks of age [10]. The local chicken ecotypes from Benin also had significant increases in live weight, hot and cold carcass weight, as well as that of different cuts with age, where 24-week-old chickens had higher values than that of 20-week-old chickens. In Côte d’Ivoire, similar results were reported in local breed chickens [11] (Forest, Savannah) from 12 to 22 weeks of age. It is beyond 24 weeks of age that local chickens in Benin reach maturity and their body weight increases slightly over a longer period [12]. Moreover, similar results have been reported in other poultry species. For example, in Muscovy ducks, it has been reported that at slaughter, the live, hot carcass, cold carcass, and pieces such as wishbone, wings, and thigh-pilon weights, and the weight of different components of the fifth quarter increased with the age of the birds [13].

Regarding the effect of sex, the live, hot carcass, wing, thigh, and tail weight of males were significantly higher than those of females. This result confirms the dimorphism between males and females, males are taller and larger than females. Similar results had been reported in several avian species in different studies conducted on local poultry populations of the species *Gallus gallus*, such as the effect of sex on the weights of the different components of the carcass [1, 10, 14, 15]. Other authors have shown that in local chicken breeds, the females generally grew slower than males and rarely reached 1 kg of live weight at 20 weeks of age compared to the 1.2 kg of the roosters [16–19]. A similar effect was observed in Muscovy ducks in South Benin [13].

### Variation in meat pH of Goliath chickens with age at slaughter and sex

In this study, the pH of the brevis and thigh-pilon muscles decreased as the age of the chickens increased from 12 to 20 weeks. Meat acidity increased over this aging period. This could be explained by an increase in muscle glycolytic metabolism with age and probably an increased glycogen reserve compared to 12-week-old chickens. This result trend is consistent with the observation of De Maeseneire *et al*. [20], that reported that the pH of 56-day-old slow-growing broilers was higher than that of 70-day-old birds. Another study also observed this age effect that the pH of 337-day-old spent laying hens was significantly lower than that of 220-day-old birds [21].

In contrast, it was recorded that meat pH values were higher at l, 4, 8, 12, and 24 h in 24- and 28-week-old local chickens than in 20-week-old chickens in a study conducted by Tougan *et al*. [9]. A progressive increase in pH with age was also observed in Muscovy ducks [22]. The differences between these latter results and those of this study could be explained by differences in the age periods considered and in the genetic types. Ante-mortem conditions can also vary rapidly and affect the pH of the meat. In our study, the pH did not vary with the sex of the chicken. This result is consistent with that observed in Peking ducks by Onk *et al*.[23]. In contrast, higher pH values in males than in females of local chickens from South Benin have been repeatedly recorded [1, 14, 15]. Similarly, the pH of the wishbone in males was significantly higher than that in females was noted in Muscovy ducks [21].

### Age- and sex-specific characteristics of water-holding capacity

The water-holding capacity of the thigh and tail assembly of 12-week-old chickens was higher than that of 20-week-old chickens. This could be explained by the structure of the bird’s meat before slaughter age. Indeed, water is mainly retained in the meat by myofibrillar proteins through capillarity [24]. Meat with good water retention capacity, limits weight loss during storage and processing during cooking [25]. Therefore, chicken meat from 20 weeks of age had better processing and storage ability than that from 12 weeks. In addition, this study showed that the wishbone of females had a higher water-holding capacity than that of males. However, other studies did not find any difference in the water-holding capacity by sex of local chickens [14].

### Meat color of Goliath chickens by age at slaughter and sex

The brightness and redness index of the sternum muscle of 20-week-old chickens was significantly higher than those of 12-week-old chickens. The meat of the sternum of the older chickens was, therefore, a brighter red and more oxygenated. The redness and brightness of the meat found in 20-week-old chickens compared to 12-week-old chickens in this study appeared to change over time. Other studies observed that luminance and redness index decreased significantly with age when they studied 20-, 24-, and 28-week-old cockerels [10]. In Muscovy ducks, it was also observed that ducks older than 10 months had darker muscles than younger ducks [22]. The red index and hue of the wishbone and brightness of the thigh and tail assembly of females were lower than those of males. Overall, the meat of the Goliath chickens in this study was darker in males than in females.

### Sensory characteristics of Goliath chicken meat as a function of age at slaughter and sex

The meat of the wishbone of 20-week-old Goliath chickens had an increased juiciness or sensation of juice release upon chewing than that of 12-week-old chickens. This variation in the juiciness is related to the amount of free water in the meat and variation in meat content. Fat content also influenced the meat flavor of chickens as a function of age. Thus, it is possible to estimate the juiciness of the meat from its fat content and water-holding capacity. Our results are in accordance with studies that reported that 87-day-old guinea fowl meat is less juicy than 101-day-old meat [26]. It was also reported that meat from older chickens was more appreciated by customers [27]. Furthermore, the sensory quality of Goliath chicken meat did not differ by gender. Thus, the determinants of sensory meat quality attributes studied did not vary by gender. These results are in agreement with those of other studies that found similar results [14, 15]. However, a later study observed that stronger meat flavor was obtained in females than in males when they all underwent a 2 h transport stress before slaughter [1].

On the other hand, meat from males is generally considered to be tougher than that of females [28, 29]. This difference is related to the higher collagen content in the meat of males because of an increase in testosterone that increases total collagen [25, 30]. These results are inconsistent with our results.

## Conclusion

This study on the carcass characteristics and technological and organoleptic quality of Goliath chicken meat demonstrates that meat quality varies according to the age at slaughter and sex. The pH of the wishbone muscles, thigh, and tail, as well as the water retention capacity decreases with the increasing age of the chickens. Overall, sex does not influence the pH and water retention capacity of Goliath chicken meat. The meat of the wishbone of Goliath chickens becomes a brighter red as they grow from 12 to 20 weeks of age. In addition, the meat of the older birds is juicier and more flavorful. The meat of Goliath chickens is redder in the sternum and more yellow in the thigh in males than in females. The production costs that are associated with raising Goliath chickens for 20 weeks need to be investigated.

## Authors’ Contributions

AGACA: Designed and performed the study, interpreted the results, and wrote the first version of the manuscript. AGB: Analyzed data and Revised the manuscript. KT: Drafted the manuscript. YA and IYAK: Designed the study, interpreted the results, and revised the manuscript. All authors have read and approved the final manuscript.
